# Baseline profiles of auditory, vestibular, and visual functions in youth tackle football players

**DOI:** 10.2217/cnc-2019-0008

**Published:** 2020-01-14

**Authors:** Travis White-Schwoch, Jennifer Krizman, Kristi McCracken, Jamie K Burgess, Elaine C Thompson, Trent Nicol, Nina Kraus, Cynthia R LaBella

**Affiliations:** 1 Auditory Neuroscience Laboratory & Department of Communication Sciences, Northwestern University, Evanston, IL 60208, USA; 2 Division of Orthopaedic Surgery & Sports Medicine, Ann & Robert H Lurie Children’s Hospital of Chicago, Chicago, IL 60611, USA; 3 Now at Emory University School of Medicine, Atlanta, GA 30322, USA; 4 Departments of Neurobiology and Otolaryngology, Northwestern University, Evanston, IL 60208, USA; 5 Department of Pediatrics, Feinberg School of Medicine, Northwestern University, Chicago, IL 60611, USA

**Keywords:** clinical testing, concussion, frequency-following response, sensory systems

## Abstract

**Aim:**

Neurosensory tests have emerged as components of sport-related concussion management. Limited normative data are available in healthy, nonconcussed youth athletes.

**Patients & methods/results:**

In 2017 and 2018, we tested 108 youth tackle football players immediately before their seasons on the frequency-following response, Balance Error Scoring System, and King-Devick test. We compared results with published data in older and/or and nonathlete populations. Performance on all tests improved with age. Frequency-following response and Balance Error Scoring System results aligned with socioeconomic status. Performance was not correlated across neurosensory domains.

**Conclusion:**

Baseline neurosensory functions in seven 14-year-old male tackle football players are consistent with previously published data. Results reinforce the need for individual baselines or demographic-specific norms and the use of multiple neurosensory measures in sport-related concussion management.

## Lay abstract

Previous studies have shown that tests of neurosensory function, including hearing, balance, and vision, tend to be abnormal in children and adults with a sports-related concussion. Little data are available on how youth athletes perform on these tests when they are healthy. Here we report male youth football players’ preseason performance on three tests of neurosensory function. Performance at baseline is generally consistent with previous reports in older children and/or nonathlete populations, supporting the use of these assessments when evaluating an athlete for a sports-related concussion. However, performance varies with age and socioeconomic status, reinforcing the need for an individual preinjury baseline or careful, demographic-specific norms for comparison. Performance was not correlated across neurosensory domains, supporting the use of multiple measures in concussion evaluation and management.

Neurosensory functions are receiving increasing attention as important outcome domains to consider in sport-related concussion (SRC) management [1–5]. An emerging concept is that tests that stress a sensory system tend to reveal impairments in individuals with an SRC.

The auditory, vestibular, and visual systems have received particular attention. In the auditory domain, children who experience prolonged post-SRC symptoms tested in a tertiary-care sports medicine clinic exhibit difficulty hearing in complex listening environments and poor neurophysiological responses to speech [4,5]. A history of SRC is also associated with diminished neurophysiological responses to speech in healthy, asymptomatic collegiate football players [6]. In the vestibular domain, children with SRCs seen in tertiary-care sports medicine clinics exhibit difficulties with balance [1,7,8]. In the visual domain, slowed processing speed and difficulty coordinating information between the eyes have been documented in children with SRCs seen in a tertiary-care sports medicine clinic [2], adults seen in trauma and neurosurgery settings [3], and adult pugilists [9].

In all three domains, there is evidence that the peripheral sensory organs are intact and functioning normally, but that CNS circuits responsible for integrating and efficiently processing information are impaired. There is also evidence that neurosensory abnormalities augur the severity of an SRC; Master *et al.* [10], for example, reported that visual and/or vestibular dysfunctions predict prolonged recovery in children with a concussion (sport-related or otherwise) tested in a tertiary-care sports medicine clinic. These findings suggest that relatively simple neurosensory tests can provide a ‘window’ into neurologic functions.

Neurosensory tests are thought to offer an improvement over symptom reports, which can be unreliable (i.e., patients inflating or minimizing their symptom loads) and nonspecific (such as a headache). In fact, some patients may report concussion symptoms and/or perform abnormally on concussion tests even when they are healthy. For example, in a study of over 30,000 high school student athletes, Iverson *et al.* [11] showed that girls reported higher concussion-like symptoms even though none had been diagnosed with a concussion in the preceding 6 months. Boys with a history of a concussion, but who had recovered, also exhibited higher symptom rates. This has, in part, motivated the use of neurosensory tests that can provide objective signs. Still, these tests have their limitations, including individual variations and potential learning effects, complicating their interpretation – especially in the absence of a baseline. For example, Corwin *et al.* [12] administered vestibular and visual tests used in concussion evaluation to children presenting to the emergency department with non-neurologic complaints; about one quarter of the children failed one or more components of these tests.

The opposite situation is also a complicating factor in SRC assessments. Athletes are typically motivated to return to sports as quickly as possible, which means they might conceal SRC symptoms to speed up their return. For example, McCrea *et al.* reported that only 47.3% of a sample of high school varsity football players reported their SRCs, among whom 41% said they failed to report for risk of being withheld from competition [13]. Likewise, there is concern that athletes can intentionally underperform on baseline tests (‘sandbagging’) to forestall risk that potential head injuries will show impaired performance by comparison on postinjury assessments [14].

## Evaluating auditory processing: the frequency-following response

One novel dimension of this research is the focus on auditory processing in children at risk for an SRC. The frequency-following response (FFR) is an objective electrophysiological test of auditory processing that involves measuring neurophysiological responses to a speech syllable. It is measured noninvasively with three scalp electrodes and is thought to be generated predominantly by the auditory midbrain [15,16].

A major advantage of the FFR is that the response is recorded passively – patients may sleep or watch a movie during testing and the response is unaffected [17]. This makes it an objective approach to evaluate auditory–neurophysiological function because it forestalls concerns about ‘sandbagging’ or malingering. Additionally, responses are recorded automatically and generally analyzed with computer algorithms, meaning the FFRs interpretation does not rely on subjective judgments by testers. A final major advantage of the FFR is that several, noncorrelated measures may be extracted from a single response, meaning it indicates several aspects of auditory processing [18]. Distinct clinical groups (children with concussions, individuals with language disability, individuals with autism spectrum disorder) each exhibit signature patterns of selective disruption, making the FFR a sensitive and specific marker of auditory processing in clinical populations [19,20].

Several studies provide normative and apposite psychometric data for the FFR; germane to this study, Skoe *et al.* provide norms in 586 normal-hearing individuals from ages 0 to 73 years [21] and Krizman *et al.* provide sex-specific norms in 516 preschoolers, adolescents, and young adults. While less data are available on the test–retest reliability of the FFR, studies suggest that it is in the moderate-to-good range [22,23]. With respect to concussion assessments, Kraus *et al.* [5] measured FFRs in 20 children diagnosed with a concussion compared with 20 controls, and reported that an objective algorithm incorporating multiple measures derived from the FFR had a sensitivity and specificity of 0.9 and 0.95, respectively, in identifying concussion and control cases.

## Clinical applications of neurosensory tests

Several authors have suggested that these tests could be a useful component of SRC diagnosis and management [1–5]. Yet there remain knowledge gaps in previously published studies, such as a lack of normative data in younger age groups (i.e., below high-school level). For tests where normative data are available, these norms were typically developed in a mixed sample where the proportion of athletes and nonathletes is unknown [21]. There are some data regarding baseline values for these tests in adolescent football players. For example, Munce *et al.* reported that 15 high school-aged, male football players did not show any baseline deficits on the King-Devick test (KD), ImPACT, and a test of postural stability, and that scores improved slightly when ten of them were retested after their season [24].

Thus, before neurosensory tests can be incorporated into routine SRC assessments, it is necessary to understand how healthy young athletes perform on them. To this end, we tested youth tackle football players before their season began on the FFR, the Balance Error Scoring System (BESS; a vestibular test of postural stability), and the KD test (a test of oculomotor function).

There were three specific goals of our study:

- To characterize young male athletes' baseline performance on neurosensory tests that typically show acute disruptions in concussed athletes, including in relation to previously published data on older and nonathlete populations
- To identify factors that account for individual differences in their baseline performance
- To test for correlations in performance among three domains of neurosensory function

## Methods

All study procedures were approved in advance by the Institutional Review Board of Ann & Robert H. Lurie Children’s Hospital of Chicago. Either parents or legal guardians provided written consent; children ages ≥12 years also provided written assent, while children ages 11 years and under provided verbal assent.

### Recruitment

In August 2017 and August 2018, all players in an urban youth tackle football program serving males aged 7–14 years of age were invited to participate in a study on concussion outcomes in youth sports (N ≈ 200). Exclusion criteria were a diagnosed hearing loss, epilepsy, or developmental disability, which were screened via a written questionnaire that parents completed. All subjects passed a hearing screening (clear otoscopies, normal distortion-product otoacoustic emosions, and normal auditory brainstem responses).

A few days before the start of the season, enrolled players completed tests of auditory, vestibular and visual functions. These sessions were conducted prior to any team physical activities and were scheduled to coincide with preparatory activities such as parent orientation sessions and equipment pickup. There were three to four testing sessions in each year, including 1 weekend day and 2–3 weekday evenings.

Testing sessions were conducted in a field house next to the football field. In 2017, a multipurpose room with a floor-to-ceiling room divider was used. One-half of the partitioned room was dedicated to BESS and KD testing, whereas the other half was dedicated to FFR testing. The room was kept at a quiet volume to facilitate testing. Families checked in, consented and completed paperwork in the hallway outside the multipurpose room. In 2018, the multipurpose room was again used for BESS/KD testing and a quiet classroom across the hall was used for FFR testing.

**Figure 1.**
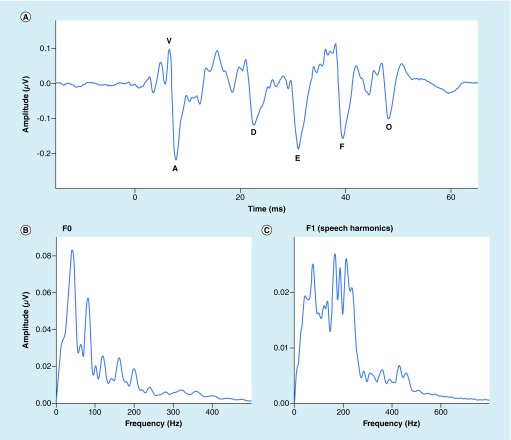
Pictures of testing. Subjects participating in the frequency-following response, Balance Error Scoring System, and King-Devick test, respectively, are shown in the multipurpose room.

Photographs of the tests and environment are shown in Figure 1.

### Auditory testing: FFRs

We measured FFRs, which predominantly reflect synchronous neural firing in the auditory midbrain. FFRs were collected by experienced electrophysiology researchers. FFRs were elicited to the speech-like sound /d/, a five-formant, 40-ms sound constructed in a Klatt-based synthesizer (SenSyn, Sensimetrics Corporation, MA, USA). Responses were recorded by a Bio-Logic Navigator Pro System (Natus; CA, USA). FFRs were measured in a vertical montage with three Ag–AgCl electrodes (Cz active, Fpz ground, A2 reference). Stimuli were delivered to the right ear via insert earphones (ER-3’s) at 80-dB sound pressure level in alternating polarities. Two runs of 3000 trials were presented, with online artifact rejection at ±23.8 μV. Responses were bandpass filtered from 100 to 2000 Hz (2nd-order Butterworth) epoched in a 75-ms time window with stimulus onset set to 0 ms and a 15.8 nonstimulus preperiod. For all but the antepenultimate-dependent variable, the envelope response was used, which is the average response of the two stimulus presentation polarities.

FFR dependent variables are:

- Neural timing. FFRs to /d/ have stereotyped peaks reflecting the response to the onset (Peaks V and A), sustained phase locking (Peaks D, E, and F), and the offset (Peak O). Latencies of each peak were identified. Smaller numbers reflect better performance (i.e., faster responses); dependent variables are referred to as ‘V,’ ‘A,’ ‘D,’ ‘E,’ ‘F’ and ‘O.’
- Response amplitude. Root mean square (RMS) amplitude of the response was calculated from 19.5 to 44.2 ms. Larger numbers reflect better performance (i.e., larger responses); the dependent variable is referred to as ‘Response amplitude.’
- Stimulus-response correlation. Each individual’s FFR was cross-correlated with the stimulus waveform. The maximum correlation within one period of the F0 was determined and converted to a Fisher’s z correlation coefficient. Larger numbers reflect better performance (i.e., more accurate responses); the dependent variable is referred to as ‘stimulus-response correlation.’
- F0 response. A fast Fourier transform was applied to the envelope response from 19.5 to 44.2 ms (2 ms Hanning window). Total amplitude of the spectrum from 75 to 175 Hz was calculated. Larger numbers reflect better performance (i.e., larger responses); the dependent variable is referred to as ‘F0.’
- F1 response (speech harmonics). A fast Fourier transform was applied to the fine structure response using the same parameters as for the F0 response. To obtain the fine structure response, responses to alternating polarities were subtracted. Total amplitude of the fine structure spectrum from 175 to 750 Hz was calculated. Larger numbers reflect better performance (i.e., larger responses); the dependent variable is referred to as ‘F1.’
- Response consistency. Two subaverages of the response, each comprising 2000 sweeps, are correlated and converted to a Fisher’s z correlation coefficient. Larger numbers reflect better performance (i.e., a more stable/less variable response); the dependent variable is referred to as ‘response consistency.’
- Prestimulus amplitude. RMS amplitude of the prestimulus region of the response (-15 to 0 ms) was calculated. This time region corresponds to the silent gap between stimulus presentations and provides a level of noise in each response. Smaller numbers reflect better performance (i.e., a lower level of nonstimulus-evoked neural activity); the dependent variable is referred to as ‘prestimulus amplitude.’

### Vestibular testing: BESS

BESS testing was performed by one of three certified athletic trainers or an advanced practice nurse experienced in its administration and the care of young athletes with SRCs. Each had been trained and met inter-rater reliability levels following procedures described in a previous study [25]. Three positions were tested: feet touching side-by-side, single-leg stance on the nondominant leg, and heel-to-toe stance with the dominant foot in front. The dominant leg was determined by asking subjects which foot they would use to kick a ball. Subjects were instructed to close their eyes, place their hands on their hips and hold each pose for 20 s each on a firm floor surface and a 6-cm thick foam pad (Airex Balance-Pad Elite; Airex AG, Sins, Switzerland). One error point was given each time the subject moved hands off the hips, opened eyes, stepped, stumbled, abducted, or flexed the hip more than 30°. For each trial, the maximum score was ten points. If a subject could not maintain a position for ≥5 s that trial was assigned ten points. The vestibular dependent variable was the sum of the scores from all six trials.

### Visual testing: the KD test

KD tests were administered via an iPad app by one of three certified athletic trainers or an experienced advanced practice nurse. Subjects were asked to read the numbers as quickly as possible without error, as per the KD iPad app instruction manual [26]. After subjects completed two error-free runs, the naming time of the faster run was recorded. Subjects read aloud single-digit numbers on a practice card and then on two (subjects aged 7–9) or three (subjects ages 10+) test cards. The visual dependent variable was the average reading time per card.

### Statistical analyses

Data analyses were organized around our three specific aims. All p-values reflect two-tailed tests at a 0.05 significance level (Bonferonni corrections for certain analyses are noted in the Results section). CIs reported are 95% intervals that were bootstrapped with 10,000 iterations. Analyses were performed in SPSS Version 25 (IBM, NY, USA).

To characterize young male athletes’ baseline performance on neurosensory tests that typically show acute disruptions in concussed athletes, descriptive analyses were run on scores on all of the tests, focusing on the range and distribution of performance.

Because age-appropriate norms were available for the FFR [21], age-corrected z-scores were calculated to compare this cohort to the normative cohort. Subjects were split into 2-year age bins (i.e., ages 7–8, 9–10, 11–12, and 13–14). Scores were computed such that positive z-scores always reflected better performance (faster latencies, larger responses, lower neural noise). Because sex effects have been found in the FFR [27], we used norms only for males.

The cohort’s mean performance was compared with the population mean (z-score = 0) using one-sided *t*-tests. The number of individuals performing more than two standard deviations (SDs) below the age-referenced norm were counted – we only expect 5% (5–6 subjects) performing in this range and tested whether more or fewer subjects than expected fell into this range with Fisher’s exact test. The number of individuals performing within one SD of the population mean was also computed (66% expected, or 71–72 subjects) and we tested whether more or fewer subjects than expected fell into this range with a chi-squared test.

Norms for males in this age range were not available for the BESS or KD, but the cohort’s performance was compared qualitatively with the previous literature [25,28].

To identify factors that account for individual differences in athletes’ baseline performance on neurosensory tests, we used multiple regression. Candidate factors that could account for individual differences in baseline performance were age and socioeconomic status (SES). SES was approximated by referencing each subject’s home ZIP code to US Census median income for that area [29]. We will refer to this as ‘estimated household income.’ For each dependent variable, these factors were entered into a multiple linear regression.

Finally, correlations among the tests were evaluated, controlling for age and SES.

## Results

### Subjects

In 2017, 57 subjects were recruited, six of whom were excluded for poor FFR data (artifacts more than 20% total samples on the test; see the Methods section). This left 51 subjects. In 2018, 79 subjects were recruited, 23 of whom participated in 2017. For 20 of these subjects, 2017 data were used. However, three of the 2017 subjects with poor data had usable data in 2018, and so those tests were used. Of the 59 new subjects in 2018, two had poor data and so were excluded. Details are provided in Table 1.

**Table 1.** Exclusions and subject demographics.

Demographics	2017	2018	Combined
Recruited	57	79	136
Excluded for poor FFR data (artifacts >20%)	6	2	8
Excluded due to participation in 2017	N/A	20	20
Subjects included	51	57	108
Age (mean [SD])^†^	12.0 (1.79)	11.2 (1.5)	11.6 (1.7)
Estimated household income (mean [SD])^†^	US$69,662 (US$17,992)	US$72,656 (US$16,230)	US$71,309 (US$17,023)

† Means and SDs are only shown for subjects included in the analyses.

FFR: Frequency-following response; N/A: Not applicable; SD: Standard deviation.

The 2017 cohort was slightly older, on average, than the 2018 cohort (by about 9 months; t(106) = 2.31, p = 0.023, d = 0.48; see Table 1). The 2017 and 2018 cohorts were similar with respect to estimated household income (t[98] = 0.87, p = 0.38; see Table 1).

The combined, final dataset represented 108 children. The mean age was 11.6 years (SD, 1.7 years, range, 7.3–14.0 years; see Table 1). ZIP codes were available on 100 children. The mean estimated household income was US$71,309 (SD, US$17,023, range, US$33,959–107,811; see Table 1). Age and estimated household income were not correlated (r[98] = 0.13; p = 0.18).

### Aim 1: to characterize young male athletes’ baseline performance on neurosensory tests & compare to previously published data

Means, ranges and standard deviations for all measures are reported in Table 2.

**Table 2.** Descriptive statistics.

Dependent variable	N	Mean (95% CI)	SD	Range
V	108	6.63 (6.59–6.68)	0.23	6.03–7.28
A	108	7.71 (7.65–7.770)	0.23	6.78–8.78
D	108	22.64 (22.53–22.76)	0.33	21.53–25.62
E	108	31.02 (30.94–31.10)	0.58	29.95–32.2
F	108	39.48 (39.39–39.56)	0.44	38.53–40.95
O	108	48.19 (48.08–48.29)	0.45	44.95–49.87
Response amplitude	108	0.104 (0.099–0.109)	0.0260	0.044–0.1934
Stimulus–response correlation (z)	108	0.234 (0.222–0.245)	0.0606	0.0282–0.3934
F0	108	0.0568 (0.0538–0.0599)	0.0163	0.0072–0.045
F1	108	0.0194 (0.0181–0.0207)	0.0072	0.0134–0.054
Response consistency (z)	108	1.12 (1.06–1.17)	0.3066	-0.1314 to 1.4863
Prestimulus amplitude	108	0.032 (0.030–0.034)	0.0091	0.044–0.1934
KD	103	20.87 (19.89–21.87)	5	11.6–33.65
BESS firm	97	8.23 (7.35–9.15)	4.51	0–20
BESS foam	97	16.76 (15.88–17.64)	4.38	7–30
BESS total	97	24.99 (23.46–26.53)	7.63	7–44

BESS: Balance error scoring system; KD: King-Devick test; SD: Standard deviation.

**Figure 2.**
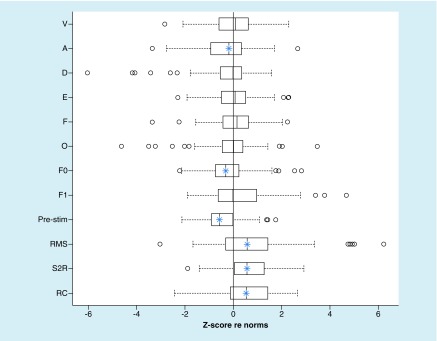
The frequency-following response. The grand-average frequency-following response is shown in the time **(A)** and frequency **(B/C)** domains. Stereotyped peaks are labeled in **(A).** The frequency bins used to calculate F0 and F1 (speech harmonics) amplitudes are shown in **(B)** and **(C)**, respectively. F0 was calculated on the frequency-following response to the envelope (responses to alternating polarities added) and F1 on the response to the temporal fine structure (responses to alternating polarities subtracted).

**Figure 3.**
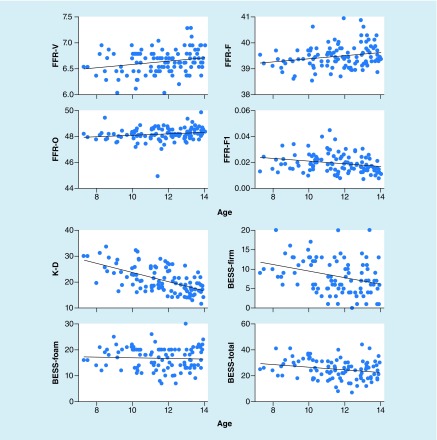
Norm-referenced performance on the frequency-following response. Scores on FFR measures were converted to age-referenced z-scores (re-norms developed in nonathletes). The population mean has a z-score of 0. The boxplots show the cohort’s performance re-norms on each FFR measure. Shaded regions represent ±2.5 standard deviations. Blue asterisks indicate measures where the cohort’s performance was significantly worse (left of zero line) or better (right of zero line) than normal (one-sided t-test). FFR: Frequency-following response.

The grand average FFR in the time and frequency domains is shown in Figure 2. Age-normed z-scores are presented in Figure 3 and percentiles are presented in Table 3. Generally speaking, the cohort performed similarly to norms. The sample’s z-scores were slightly below the population mean on A latency, F0 amplitude, and the prestimulus amplitude, indicating that this cohort performed slightly worse than norms. However, response amplitude, stimulus–response correlation, and response consistency were all better than the norms. Thus, the cohort performed higher than the norm on as many tests as they performed lower than the norm.

**Table 3.** Norm-referenced percentiles and proportion falling outside normal limits on frequency-following response measures.

Dependednt variable	Mean	Median	Range	^#^±1 SD^†^	^#^≤ -2 SD^‡^
V	50.23	53.48	0.23–98.98	79 (73.15%)	3 (2.78%)
A	42.69^¶^	42.87	0.04–99.36	77 (71.30%)	4 (3.70%)
D	47.47	49.61	0–94.41	89 (82.41%)^§^	6 (5.56%)
E	51.02	56.08	0.04–98.78	87 (80.56%)^§^	1 (0.93%)
F	53.06	56.08	0.04–98.78	87 (80.56%)^§^	2 (1.85%)
O	47.87	50.45	0–99.97	84 (77.78%)	5 (4.63%)
F0	42.58^§^	37.73	1.35–99.77	78 (72.22%)	2 (1.85%)
F1	52.41	49.52	2.96–100	66 (61.11%)	0 (0%)
Prestimulus amplitude	35.94^#^	28.35	1.67–96.1	79 (73.15%)	1 (0.93%)
Response amplitude	64.48^#^	71.77	0.12–100	58 (53.7)^§^	1 (0.93%)
Stimulus–response correlation	68.55^#^	71.51	2.91–99.83	67 (62.04%)	0 (0%)
Response consistency	67.15^#^	70.36	0.74–99.63	64 (59.26%)	1 (0.93%)

† It is expected that 66% of the population falls within this range. Expected and actual distributions were compared with χ^2^ tests.

‡ It is expected that 2.5% of the population performs within this range. Expected and actual distributions were compared with the Fisher’s exact test.

§ p < 0.05.

¶ p < 0.01.

# p ≤ 0.001.

SD: Standard deviation.

Only the differences between norms and this cohort’s prestimulus amplitude, response amplitude, stimulus–response correlation, and response consistency met the Bonferonni-corrected threshold of p < 0.004. The proportion performing within one SD of the norm was slightly higher than expected for D and F latencies and slightly lower than expected for the prestimulus noise (χ^2^ tests, all p < 0.05; Table 3).

Importantly, and despite these small variations in the population’s median performance, the number of children falling 2+ SDs below norms was ≤6 (Table 3) – exactly how many would be predicted by the normal distribution. In no case were there more children performing below normal than expected (Fisher’s exact test; all p > 0.25).

As expected, BESS performance was worse on the foam surface than the firm surface, similar to previous studies. On average, subjects made twice as many errors in the foam condition than the easier firm condition (about 8 on firm and about 16 on foam; t[96] = 18.36, p < 0.001). These scores are higher than those reported by a previous study of males ages 10–17 years (8 firm/15 foam errors in our study vs 5 firm/12 foam errors previously) [25].

For the KD test, it took subjects, on average, 20 s to complete the task. While norms have not been published, a previous study used linear regressions to estimate average naming time per card on this task [28]. Approximately 20 s per card matches estimated performance for the age range in our study.

### Aim 2: to identify factors that account for individual differences in young athletes’ baseline performance

Multiple linear regressions were used to test for associations between neurosensory tests, age and estimated household income. Full regression results are reported in Table 4, with the total variance explained by each model for each dependent variable, along with *post-hoc* correlations. Here, we summarize only the significant correlations.

**Table 4.** Results of regressions.

Measure	Overall (*R*^2^)	Age (*β*)	Income (*β*)
V	6.1%^‡^	0.033^‡^	-0.012
A	2.3%	0.027	-0.015
D	2.9%	0.046	0.031
E	3.1%	0.045^†^	0.002
F	7.0%	0.062^‡^	-0.043
O	4.9%^†^	0.058^‡^	-0.002
F0	3.3%	-0.001	0.002
F1	4.3%	-0.001^‡^	<0.001
Prestimulus amplitude	4.2%	<0.001	<0.001
Response amplitude	1.4%	-0.001	0.002
Stimulus–response correlation	1.4%	-0.006^†^	0.004
Response consistency	6.8%	0.021	0.038^‡^
KD	34%^§^	-1.677^§^	-0.148
BESS firm	14.1%^§^	-0.805^§^	-0.459^†^
BESS foam	7.1%^‡^	-0.084	-0.651^‡^
BESS total	12.0%	-0.890^‡^	-1.110^‡^

† p < 0.1.

‡ p < 0.05.

§ p < 0.001.

BESS: Balance error scoring system; KD: King-Devick test.

**Figure 4.**
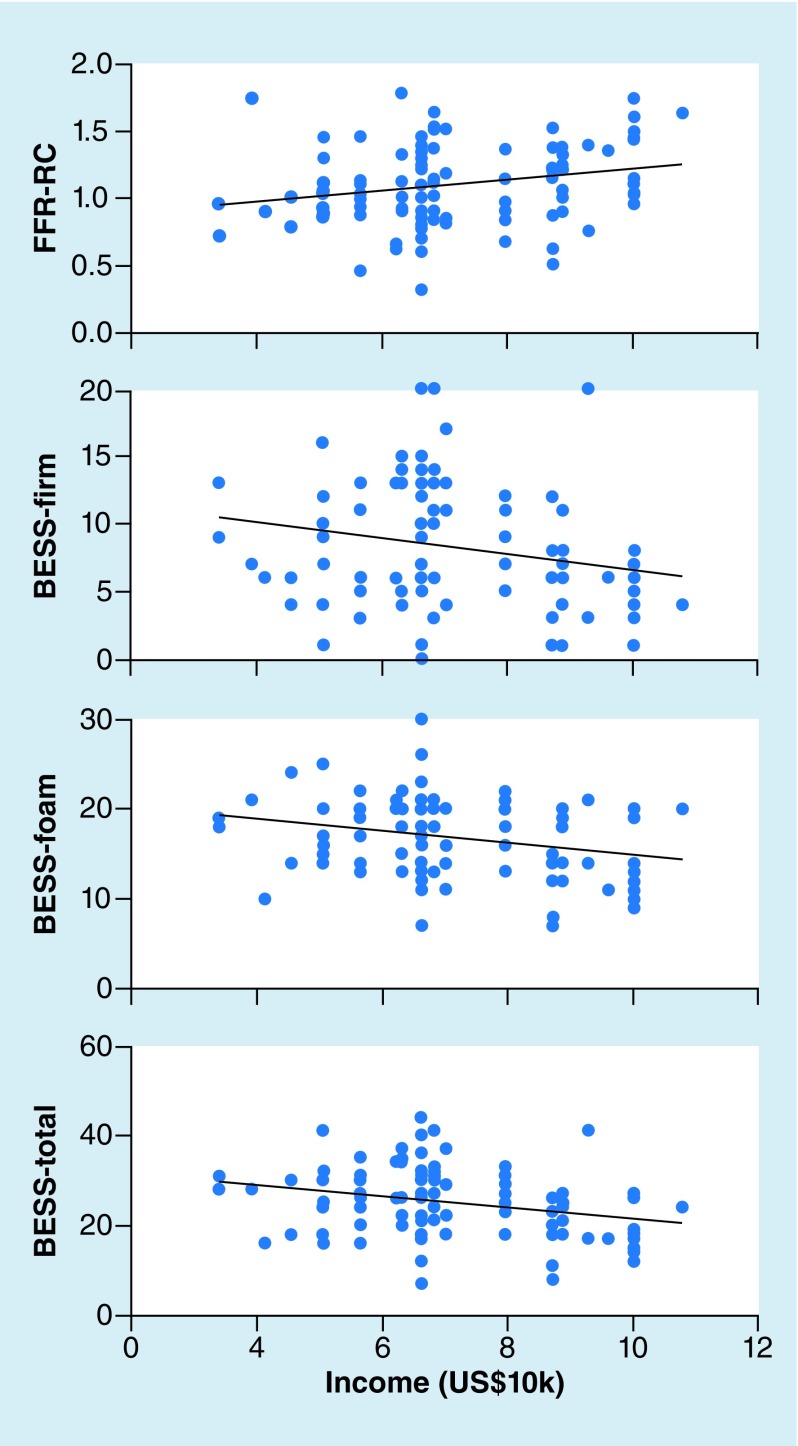
Correlations between age and performance on neurosensory tests. Age was correlated to performance on all measures. Increased age was associated with slower frequency-following response V, F, and O latencies. Increasing age was also associated with better performance on the King-Devick test and the BESS. BESS: Balance error scoring system; FFR: Frequency-following response.

Age was significantly correlated with (see Figure 4 for scatterplots):

- FFR waves V, F, and O latencies, which were slower in older subjects. For Wave V, each year was associated with a 0.033-ms later response (*β* = 0.033, SE = 0.014, *t* = 2.466, p = 0.015). For Wave F, each year was associated with a 0.62-ms later response (*β* = 0.062, SE = 0.026, *t* = 2.357, p = 0.020). For Wave O, each year was associated with a 0.063-ms later response (*β* = 0.058, SE = 0.026, *t* = 2.221, p = 0.029).
- FFR F1 amplitude (speech harmonics), which was smaller in older subjects. Each year was associated with a 1.0-nV decrease in amplitude (*β* = -1.0, SE <1.0, *t* = 2.071, p = 0.041).
- KD performance. Each year was associated with 1.68-s faster performance on the test (*β* = -1.677, SE = 0.250, *t* = 6.705, p < 0.001).
- BESS performance, which was driven by scores on the firm condition. For the firm condition, each year was associated with 0.81 fewer errors (*β* = -0.805, SE = 0.258, *t* = 3.119, p = 0.002) and for total scores, each year was associated with 0.89 fewer errors (*β* = -0.890, SE = 0.437, *t* = 2.253, p = 0.013).

**Figure 5.**
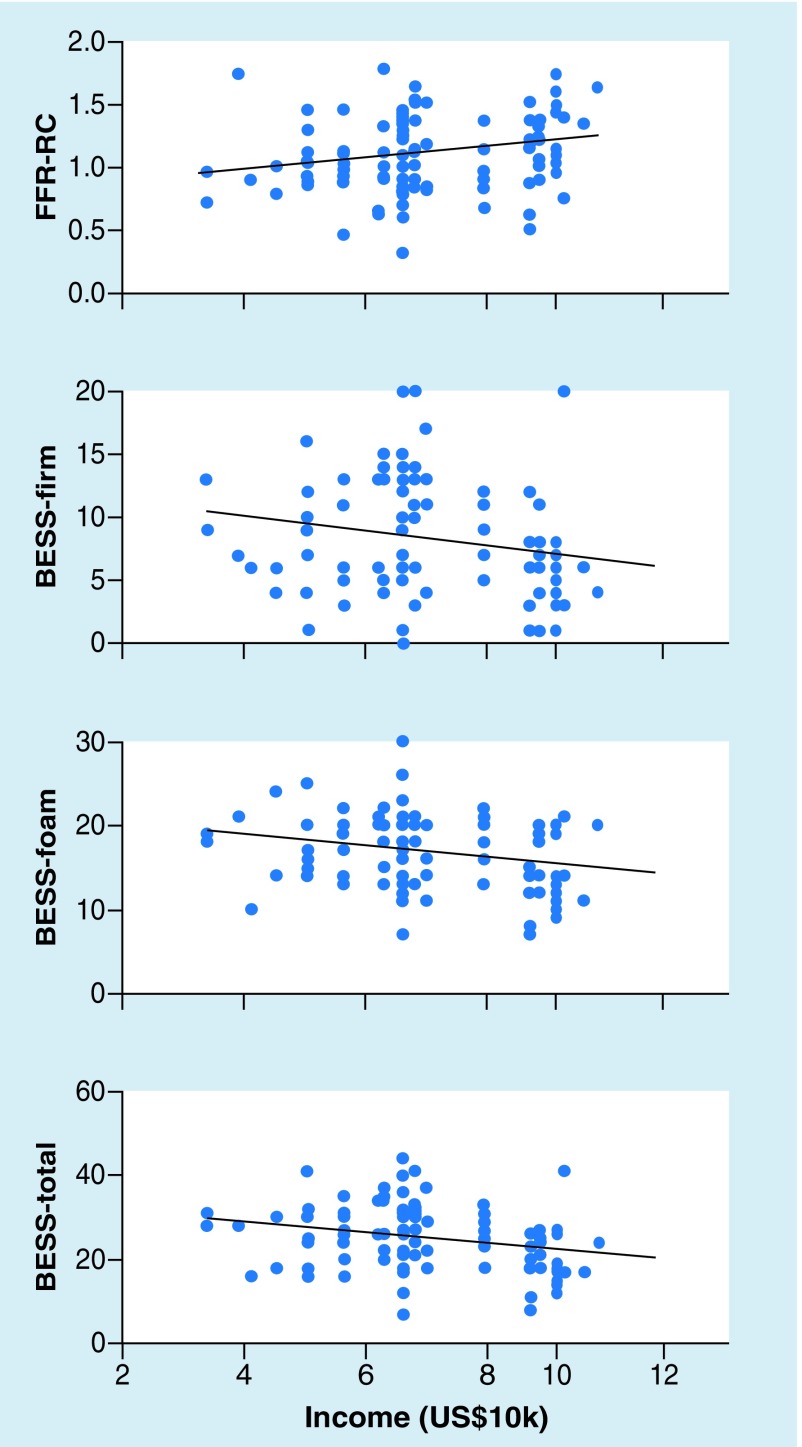
Correlations between estimated income and performance on neurosensory tests. Area-estimated income, a proxy for socioeconomic status, was correlated with performance on all measures. Higher estimated incomes were associated with more consistent frequency-following responses and better performance on the Balance Error Scoring System. BESS: Balance error scoring system; FFR: Frequency-following response.

Estimated household income was significantly correlated with (see Figure 5 for scatterplots):

- FFR response consistency, with each US$10,000 jump in income associated with response consistency scores that were 0.038 Fisher’s *z* units higher (*β* = 0.038, SE = 0.017, *t* = 2.173, p = 0.032).
- BESS performance, which was driven by scores on the foam condition. For the foam condition, each US$10,000 income was associated with 0.66 fewer errors (*β* = -0.661, SE = 0.259, *t* = 2.555, p = 0.012) and for total scores, each US$10,000 income was associated with 1.1 fewer errors (*β* = -1.110, SE = 0.438, *t* = 2.534, p = 0.013).

### Aim 3: to test for correlations among the three domains of neurosensory function

Next, we tested for correlations among the three test modalities (auditory, vestibular, and visual). We covaried for age and estimated income, because we identified these as factors that affect scores on two or three of the sensory domains.

As shown in Table 5, there were no correlations between domains (all r < 0.1 and all p > 0.2). The only significant correlations were among multiple features of the FFR.

**Table 5.** Correlations among all measures, controlling for age and estimated household income.

Dependent variable	V	A	D	E	F	O	F0	F1	Prestimulus amplitude	Response amplitude	Stimulus–response correlation	Response consistency	KD	BESS total
V	1.000	0.724^‡^	0.366^‡^	0.479^‡^	0.622^‡^	0.376^‡^	0.069	-0.156	0.045	-0.098	-0.085	-0.104	-0.084	-0.006
A	0.724^‡^	1.000	0.400^‡^	0.466^‡^	0.473^‡^	0.293^‡^	0.018	-0.240^†^	0.032	-0.169	-0.146	-0.045	-0.095	0.029
D	0.366^‡^	0.400^‡^	1.000	0.257^†^	0.251^†^	0.316^‡^	-0.009	-0.025	0.062	-0.063	-0.231^†^	-0.183	0.026	0.040
E	0.479^‡^	0.466^‡^	0.257^†^	1.000	0.505^‡^	0.325^‡^	0.178	-0.034	-0.011	0.012	-0.060	0.144	0.030	0.000
F	0.622^‡^	0.473^‡^	0.251^†^	0.505^‡^	1.000	0.224^†^	0.223^†^	-0.089	-0.084	0.081	-0.134	0.097	-0.021	-0.023
O	0.376^‡^	0.293^‡^	0.316^‡^	0.325^‡^	0.224^†^	1.000	0.063	-0.048	0.109	-0.031	-0.106	0.023	-0.086	-0.014
F0	0.069	0.018	-0.009	0.178	0.223^†^	0.063	1.000	0.021	0.010	0.817^‡^	-0.043	0.521^‡^	-0.137	-0.002
F1	-0.156	-0.240^†^	-0.025	-0.034	-0.089	-0.048	0.021	1.000	0.068	0.135	-0.092	0.075	0.052	-0.027
Prestimulus amplitude	0.045	0.032	0.062	-0.011	-0.084	0.109	0.010	0.068	1.000	0.147	-0.178	-0.271^‡^	-0.052	0.107
Response amplitude	-0.098	-0.169	-0.063	0.012	0.081	-0.031	0.817^‡^	0.135	0.147	1.000	-0.132	0.502^‡^	-0.053	-0.027
Stimulus–response correlation	-0.085	-0.146	-0.231^†^	-0.060	-0.134	-0.106	-0.043	-0.092	-0.178	-0.132	1.000	0.186	0.005	0.008
Response consistency	-0.104	-0.045	-0.183	0.144	0.097	0.023	0.521^‡^	0.075	-0.271^‡^	0.502^‡^	0.186	1.000	-0.093	-0.001
KD	-0.084	-0.095	0.026	0.030	-0.021	-0.086	-0.137	0.052	-0.052	-0.053	0.005	-0.093	1.000	0.006
BESS total	-0.006	0.029	0.040	0.000	-0.023	-0.014	-0.002	-0.027	0.107	-0.027	0.008	-0.001	0.006	1.000

† p < 0.05.

‡ p < 0.01.

BESS: Balance error scoring system; KD: King-Devick test.

## Discussion

This is the first study to report baseline performance on three separate domains of neurosensory function (auditory, vestibular and visual) in a cohort of healthy male youth tackle football players. While these neurosensory domains may show deficits in a patient with an SRC, their clinical utility is limited in children without individual baseline results because previously published data for the KD and BESS do not include these younger age groups, and published norms for the FFR do not consider athlete status. Our results indicate the need to develop careful, demographic-specific norms.

We chose tests that typically exhibit acute and chronic disruption in athletes with SRCs. Prior to incorporating them into clinical care, however, it is important to understand how healthy young athletes perform. Similarly, it is important to understand factors that might influence their performance. While there were subtle differences between our cohort’s performance and previously published norms on the FFR, the cohort did not deviate dramatically from typical performance. Older age was associated with improved performance on several measures of neurosensory function. Estimated household income, a proxy for SES, was also associated with individual differences in performance on the BESS and FFR response consistency.

### Young male athletes’ performance on neurosensory tests at preseason baselines

One concern in SRC evaluations is that, even in healthy patients, there are moderate-to-high failure rates on SRC tests. This concern is easy to imagine since SRC symptoms are nonspecific. For example, headache and dizziness are also symptoms of many other medical conditions. Indeed, epidemiologic studies have shown that 19% of boys and 28% of girls report symptoms commonly seen with SRC even when they are healthy and have no history of concussion [11]. The recent focus on neurosensory measures, which provide objective signs to complement symptom evaluations, was motivated in part by the hope that there would be lower failure rates in healthy athletes. Yet, failure rates are still relatively high among some vision tests [12].

While norms are not available for the KD or BESS, they are available for FRR, so we were able to evaluate our cohort’s norm-referenced performance on the FFR. Overall, the group performed similarly to the norms. There were some measures where the group tended to perform slightly higher than norms, and others where the group tended to perform slightly worse than norms (Figure 2). These differences could be due to several factors. A chief consideration is that testing in this study was conducted in a community recreation center, with multiple electrophysiology stations in the same room. In contrast, norms were collected in a laboratory setting (sound-attenuated and electrically shielded booth). Indeed, in this cohort, responses were slightly noisier than normal, as evaluated by RMS amplitude of the prestimulus period. Yet, other measures were slightly better than the norms. It is possible that differences such as language and athletic experience account for some of this variation [30]. Norms were also referenced to 2-year age bins, which may still be too coarse in this age range (see Figure 3 & Table 3).

Nevertheless, when evaluating failure rates on FFR measures our cohort performed as expected (Table 2). That is, the number of children performing more than two SDs below the mean was exactly as would be expected under the normal distribution. This suggests that FFR norms can be applicable to new populations, at least in the context of making pass–fail judgments. While the application of FFR to concussion evaluation remains in its early stages, this does lend promise to the development of valid criteria for clinical evaluation.

While standardized norms are not available for the BESS or KD, we are able to make some general comparisons to previously published data. Our group performed slightly worse on the BESS than a previous study of older male athletes [25]. Given that BESS performance improves with age, and that our cohort included younger children, age could be a factor in these differences. The fieldhouse setting of our study may, too, account for performance differences, particularly because there might be additional distractions. Indeed, Rahn *et al.* showed that collegiate student–athletes commit more errors on the BESS in distracting settings (sidelines of football and basketball stadia) than in a quiet laboratory. Our unexpected finding of an association between estimated household income and BESS performance suggests that the diversity of SES in our subject pool might also account for some differences between our results and those previously published.

On the KD, our group performed roughly similar to males and females in a previously studied developmental paper of healthy children [28]. That paper also identified an association between performance and age, and showed that naming time improved by 1.6–1.8 s per year. In our study, performance improved 1.7 s per year, on average, suggesting our cohort performed similarly to subjects in previously published studies.

### Factors that influence performance at baseline

Another concern in SRC evaluations is that performance on many measures can be affected by factors other than the SRC itself [31]. For example, anxiety is a common symptom following a concussion, but a nonconcussed athlete with generalized anxiety disorder may report relatively high anxiety symptoms even when not concussed. Similarly, neurocognitive measures such as the ImPACT are sensitive, detailed measures of cognitive functions in the context of SRCs, but their interpretation can be complicated by factors such as learning disabilities. We considered two factors that might account for variation in baseline performance on these tests.

The first factor, age, was most commonly associated with variation in neurosensory performance. Older children had slower peak timing in the FFR, consistent with previous reports [21,32]. Additionally, older children had faster naming time on the KD and performed better on one of the BESS conditions. This suggests that age-specific norms are necessary in evaluating FFR timing, visual naming and postural stability. Similarly, if evaluating a patient for changes from baseline it may be necessary to ensure the baseline reference is relatively recent. In addition to developmental changes, we note that age may be correlated to other relevant factors, such as cumulative sports exposure. Germane to SRC evaluations is the fact that contact exposure in tackle football increases cumulatively as children get older. However, performance on neurosensory domains also improves with age. Future, longitudinal work in larger cohorts can disentangle these factors.

The second factor we considered was SES, roughly approximated by the median income in children’s home ZIP codes. Although children all came from Chicago (IL, USA), there was a wide range of incomes in their home neighborhoods. There was a small association between estimated income and the FFR consistency, consistent with previous reports [33,34]. We note, however, that consistency of the FFR has not been implicated in concussions. Still, in evaluating auditory–neurophysiological function, it is important to consider SES as a factor that might affect performance on these tests.

We also found an association between estimated income and performance on the BESS, with each US$10,000 estimated income associated with about one fewer error. This result was surprising, and we do not have a strong hypothesis to account for it. This could be due to experience-dependent and cognitive influences on vestibular functions, which may be correlated to SES. If this effect is replicated, family socioeconomic characteristics might be an important factor to consider in balance assessments. One factor that we did not account for, but might also tie into balance and/or SES, is the number of other sports subjects played. Children from higher socioeconomic backgrounds might have additional resources to participate in several sports, and to do so more frequently, which could provide additional vestibular training. This emphasizes the need to develop norms for young children that account for these multiple, inter-related factors in devising tests to evaluate SRC.

### Independence of neurosensory measures in healthy children

We showed that there were no correlations among measures of auditory, vestibular, and visual function in healthy young athletes. The independence of tests of these three domains suggests that, at least at baseline, they provide complementary pieces of information about neurosensory functions. This reinforces the need for multimodal and multidisciplinary tests in SRC evaluation and management, and to include objective physiological measures along with other signs and symptoms [35,36].

One caveat is that all of the domains were correlated with age, indicating the need to develop age-specific norms on all these measures and the importance of repeated (i.e., annual) baseline testing. It should also be pointed out that two or more of these domains may be acutely disrupted in an athlete with an SRC. Still, their independence suggests they reflect dissociable mechanisms, with each providing unique information. This hypothesis raises the prediction that the three domains could recover at different rates in athletes with SRCs, again reinforcing the concept of multimodal test batteries. Future work can test this hypothesis, in addition to testing for factors that could indicate if an athlete is at higher risk for dysfunction in one or more specific neurosensory domains.

### Limitations & future directions

A major limitation of this study is that our sample only included healthy boys. While this is representative of youth tackle football, it will be important to pursue similar studies in young female athletes. There are sex differences in auditory processing that are reflected in the FFR [27], and there may be different rates of neurodevelopment during adolescence. To our knowledge, there are no reported sex differences for the KD or BESS, but this is an important question for future studies – especially in younger children where sex differences in performance may be more prevalent. The evidence that baseline symptom reporting levels can be higher in girls than boys [11,36] motivates careful analysis of girls’ baseline performance on other concussion tests. A similar limitation of this study is that we do not have detailed information about children’s medical histories, including previous concussions, headaches, or learning problems – all of which could influence baseline performance on these tests.

Another limitation of this study is the moderate sample size. Although we tested more than 100 athletes over 2 years, the football league is one of many such leagues in the Chicago area. It will be important to replicate these results in a larger population to determine their generalizability. Similarly, given the preliminary evidence we found for SES disparities in performance on these tests, it will be important to test diverse cohorts of children on these tests, ideally with a more precise measure of SES and related factors, such as parental education level. We also did not have a detailed measure of SES. We used ZIP code, which correlates with other SES-related factors, but it only provides a rough estimate. Additionally, we did not measure participation in other sports, which may also be associated with baseline performance differences in these tests.

Finally, we note that in clinical contexts KD and BESS are conventionally considered with respect to patients’ baseline performance – that is, what is clinically meaningful is a change from baseline, not performance on a single test. Schmidt *et al.*, for example, reported that within-subject comparisons can be up to 7.6-times more sensitive to concussions than comparisons of postinjury performance to norms [37]. While it is ideal to have baseline assessments when evaluating children for an SRC, this is not always feasible, which is why we wanted to investigate children’s performance on a single test. Our observation that KD and BESS performance changes across this age range also indicates the need for annual (if not more frequent) rebaselining of young athletes.

## Conclusion

This is the first study to report baseline auditory, vestibular, and visual functions in seven 14-year-old male tackle football players. In our cohort, performance on these tests is generally consistent with previous published norms in older and/or nonathlete populations.

However, performance varied with age and SES, reinforcing the need for individual baselines or careful, demographic-specific norms for comparison. Performance was not correlated across the three neurosensory domains, supporting the use of multiple neurosensory measures in SRC evaluation and management.

**Summary points**

**Neurosensory tests of concussion**

- Tests of neurosensory function, including hearing, balance, and vision, tend to be abnormal in children and adults with a sports-related concussion. Little data are available about how healthy young athletes perform on these tests.
- Participants in a youth tackle football league performed tests of auditory (frequency-following response), vestibular (Balance Error Scoring System [BESS]) and visual function (King-Devick test).

**Baseline performance on neurosensory tests**

- Frequency-following responses were compared with published norms for nonathlete males of similar age. Overall, male football players in this study performed similarly to previously published data.
- Formal norms are not available for King-Devick test or BESS, but players in this study performed similar to published data on males of similar age.
- Increasing age was associated with improved performance on all tests.
- Higher socioeconomic status was associated with improved performance on the BESS.
- Performance was not correlated among the three tests.

**Limitations**

- Study sample was relatively small (N = 108) and only included boys.
- Did not test for differences in baseline performance as a function of medical history, such as previous concussion or diagnosed learning problem.

**Conclusion**

- Young male football players perform similarly to nonathlete peers on neurosensory tests used in concussion evaluation.
- It is important to establish demographic-specific norms for these tests.
- It may be necessary to reevaluate baselines annually during preadolescence.
- Performance was not correlated across the three neurosensory domains, supporting the use of multiple neurosensory measures in sports-related concussion evaluation and management.
